# Mean diffusivity related to rule-breaking guilt: the Macbeth effect in the sensorimotor regions

**DOI:** 10.1038/s41598-019-48654-8

**Published:** 2019-08-22

**Authors:** Seishu Nakagawa, Hikaru Takeuchi, Yasuyuki Taki, Rui Nouchi, Yuka Kotozaki, Takamitsu Shinada, Tsukasa Maruyama, Atsushi Sekiguchi, Kunio Iizuka, Ryoichi Yokoyama, Yuki Yamamoto, Sugiko Hanawa, Tsuyoshi Araki, Carlos Makoto Miyauchi, Daniele Magistro, Kohei Sakaki, Hyeonjeong Jeong, Yukako Sasaki, Ryuta Kawashima

**Affiliations:** 1 Division of Psychiatry, Tohoku Medical and Pharmaceutical University, Sendai, Japan; 2 Department of Human Brain Science, Institute of Development, Ageing and Cancer, Tohoku University, Sendai, Japan; 3 Division of Developmental Cognitive Neuroscience, Institute of Development, Ageing and Cancer, Tohoku University, Sendai, Japan; 4 Division of Medical Neuroimaging Analysis, Department of Community Medical Supports, Tohoku Medical Megabank Organization, Tohoku University, Sendai, Japan; 5 Department of Nuclear Medicine and Radiology, Institute of Development, Ageing and Cancer, Tohoku University, Sendai, Japan; 6 Creative Interdisciplinary Research Division, Frontier Research Institute for Interdisciplinary Science (FRIS), Tohoku University, Sendai, Japan; 7 Smart Ageing International Research Center, Institute of Development, Ageing and Cancer, Tohoku University, Sendai, Japan; 8 Department of Behavioral Medicine, National Institute of Mental Health, National Center of Neurology and Psychiatry, Kodaira, Tokyo, Japan; 9 Department of Psychiatry, Tohoku University Graduate School of Medicine, Sendai, Japan; 10 School of Medicine, Kobe University, Kobe, Japan; 11 ADVANTAGE Risk Management Co., Ltd, Tokyo, Japan; 12 Department of Language Sciences, Graduate School of Humanities, Tokyo Metropolitan University, Tokyo, Japan; 13 Department of Sport Science, School of Science and Technology, Nottingham Trent University, Nottingham, United Kingdom; 14 Advanced Brain Science, Institute of Development, Aging and Cancer, Tohoku University, Sendai, Japan; 15 Graduate School of International Cultural Studies, Tohoku University, Sendai, Japan

**Keywords:** Emotion, Brain

## Abstract

Guilt, a self-conscious emotion, includes self-focused role taking and also correlates with other-oriented role-taking. Excess guilt proneness might be relevant to obsessive compulsive disorders. The white matter (WM) neural correlates of the degree of guilt have not yet been determined. We hypothesized that the WM structures involved in feelings of guilt are associated with social and moral cognition (inferior parietal lobule [IPL], prefrontal cortex [PFC], and cingulate), and aimed to visualize this using diffusion MRI. We investigated the association between regional WM structures (WM volume, and fractional anisotropy, and mean diffusivity [MD]), and feelings of guilt in 1196 healthy, young students using MRI and the Guilty Feeling Scale, which comprises interpersonal situation (IPS; guilt from hurting friends) and rule-breaking situation (RBS; deontological guilt) scores. The primary novel finding presented here is that MD in the right somatosensory and motor cortices from arm to hand were positively correlated with RBS scores. Further, consistent with our hypothesis, RBS scores were positively correlated with MD in the same regions. These results would be predicted by the Macbeth effect, an obsession with dirt leading to hand-washing rituals resulting from guilt, made famous by the Shakespearian character Lady Macbeth. *“What, will these hands ne’er be clean?” William Shakespeare (Shakespeare, 1606) Macbeth*.

## Introduction

Guilt, a dysphoric feeling, involves one’s negative evaluation of specific behaviours or transgression^1^. Moreover, guilt is a self-conscious emotion, and self-reflection is necessary to induce guilt. Contrary to this self-oriented emotion, guilt-proneness is also correlated with other-oriented perspective-taking and empathic concern^2^. In brief, self-focused and other-focused role-taking might be useful for understanding the classification of guilt. People imagine themselves in others’ situation as self-focused role-taking, whereas they focus directly on others’ distress as other-focused role-taking^3^.

Deontological guilt arises from a self-judgement of having transgressed social and moral norms. Furthermore, deontological guilt motivates behaviour that is adapted to social and cultural rules^4^.

As for brain studies, a systematic review of 16 functional magnetic resonance imaging (fMRI) studies revealed guilt-related clusters of brain activation (voxel-based false discovery rate [FDR] corrected *P* < 0.05) located in the prefrontal, temporal, and parietal regions, mainly in the left hemisphere and the left dorsal cingulate cluster (voxel-based family wise error [FWE] correction *P* < 0.05)^5^. Another systematic review of 21 functional and structural imaging studies demonstrated that guilt was more likely to be associated with activity in ventral anterior cingulate cortex, posterior temporal regions, and the precuneus than were other negative moral emotions (e.g. shame and embarrassment)^6^. A previous brain grey matter (GM) anatomical study conducted by our group directly demonstrated that scores related to feelings of guilt in both interpersonal and rule-breaking situations were uniquely and negatively related to regional GM density (rGMD) in the right posterior insula (PI)^7^. To the best of our knowledge, no studies have used diffusion tensor imaging (DTI) to assess the correlation of guilt feelings and white-matter (WM) structure, including WM volume (WMV).

From a social-cognitive perspective, social knowledge is represented in the medial prefrontal cortex (mPFC)^8^. The social identity representing values in the social context was based on the ventral mPFC (vmPFC) and the posterior cingulate cortex^9^. Further, the orbitofrontal cortex (OFC) plays a known role in executive function and in controlling and correcting punishment-related behaviour^10^. Moreover, morally-relevant information is processed in the inferior parietal lobule (IPL), including the superior temporal sulcus (STS), inferior frontal gyrus (IFG), and dorsolateral PFC (DLPFC)^11^.

As we have described previously^12^, diffusion MRI is well suited to studies of WM development because changes in diffusion MRI signals may reflect alterations in myelination, a form of axonal packing that occurs with age, and may be associated with changes in emotion and cognition^13^. These tissue changes are thought to affect neural plasticity^14^. Most significantly, it has been suggested that mean diffusivity (MD), rather than radial and axial diffusivity, indicates neural plasticity^14^. For more details, please refer to the studies^13,14^.

Accordingly, we hypothesized that the WM structures that underlie feelings of guilt are also involved in social and moral cognition (IPL including STS, and PFC [mPFC, OFC, IFG, and DLPFC]). We also hypothesized that we could identify regions sensitive to the development of these capacities in young adults using diffusion MRI. Given this, the purpose of this study was to identify the WM structure associated with two types of feelings of guilt often experienced by young people. We further investigated whether the relationship between WM structures and guilt scores differed between males and females using a whole-brain level analysis, because females are often more willing to concede responsibility for misdeeds and may have more difficulty in getting rid of feelings of guilt than males^15^.

## Results

### Behavioural data

**Figure 1:**
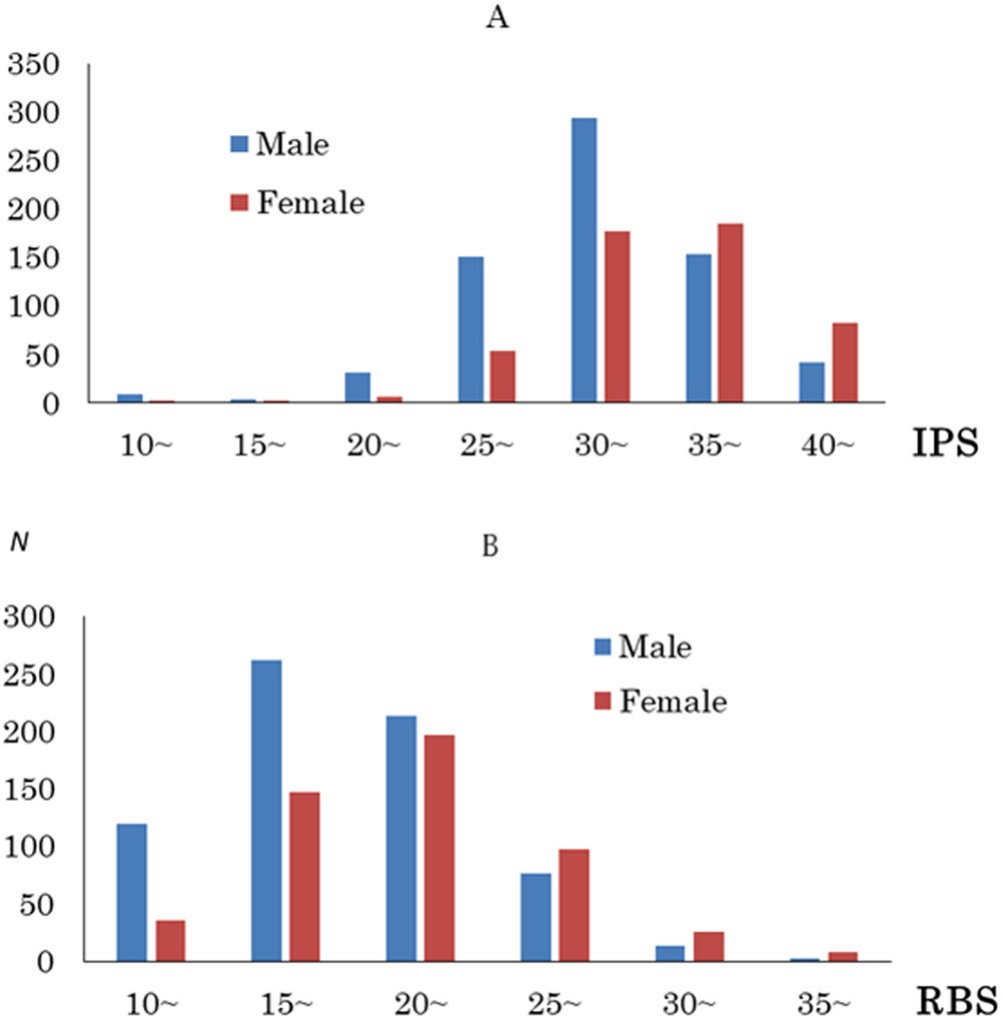
(**A**) Distribution of interpersonal situation (IPS) scores in males and females. (**B**) Distribution of rule-breaking situation (RBS) scores in males and females. Histograms show the distributions of IPS/RBS scores in males and females.

Table 1 shows the means and standard deviations (SD) for age and the scores on the Raven’s Advanced Progressive Matrix (RAPM), and guilty feeling. Figure 1 depicts the distributions of the interpersonal situation (IPS) and rule-breaking situation (RBS) scores in males and females. There were significant differences between males and females in the RAPM, IPS, and RBS scores (*P* < 0.01, one-way analysis of variance [ANOVA]).

**Table 1:** Sex differences in age, the RAPM, and IPS and RBS guilt scores (mean ± SD): one-way ANOVA results.

Measure	Total	Males (*N* = 686)	Females (*N* = 510)	*P*	*F*
Age	20.7 (1.8)	20.8 (1.9)	20.6 (1.6)	0.051	3.8
RAPM	28.5 (3.9)	28.8 (3.9)	28.1 (3.8)	0.002^*^	9.2
IPS guilt	34.0 (5.2)	32.9 (5.1)	35.6 (5.0)	<0.001^**^	85.5
RBS guilt	21.1 (5.0)	20.1 (4.8)	22.4 (5.0)	<0.001^**^	65.8

*P < 0.01, **P < 0.001.

Abbreviations: ANOVA, analysis of variance; IPS, interpersonal situation; RAPM, Raven’s Advanced Progressive Matrix; RBS, rule-breaking situation; SD, standard deviation.

### MRI data

#### Analysis of voxel-based morphometry (VBM) data

After controlling for sex, age, and RAPM scores, there were no significant positive or negative correlations between IPS or RBS scores and regional WMV (rWMV) at each voxel at a FWE-corrected threshold of *P* < 0.05, based on the threshold-free cluster enhancement (TFCE) method at the level of the whole brain.

#### Analysis of fractional anisotropy (FA) and MD data

A whole-brain multiple regression analysis that was performed after controlling for sex, age, total intracranial brain volume (TIV; total GM volume + total WMV + total cerebrospinal fluid [CSF] volume), and RAPM scores revealed no significant correlation between RBS scores (but not IPS scores) and FA.

**Figure 2:**
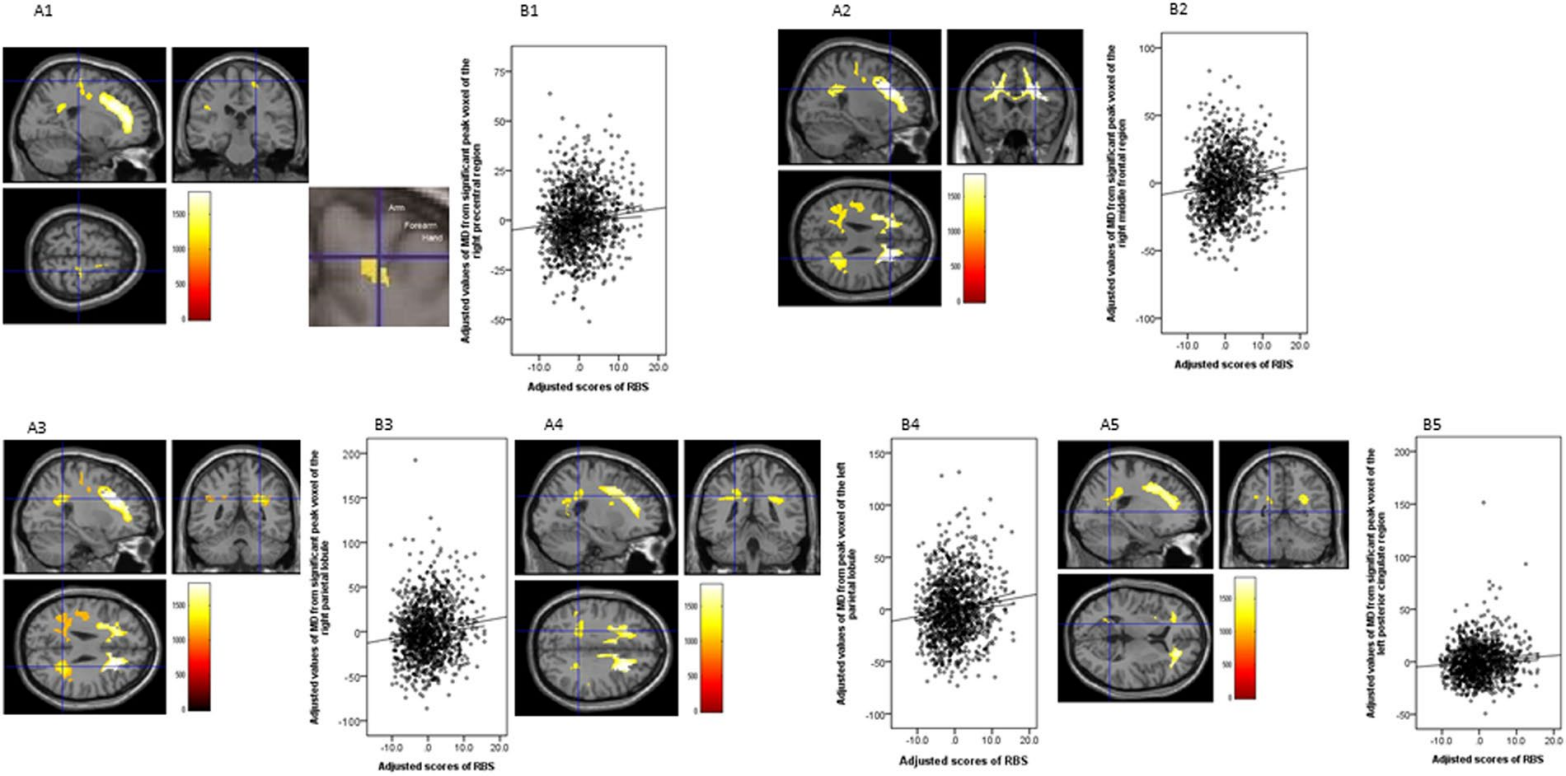
Regions correlated with mean diffusivity (MD) and rule-breaking situation (RBS) scores. The present results were determined based on a family-wise error (FWE)-corrected threshold of *P* < 0.0125 (0.05/4) with a threshold-free cluster enhancement (TFCE) based on 5000 permutations; the results were corrected at the whole-brain level. Regions showing correlations were overlaid onto a single T1 image in the SPM8 toolbox. The red-to-yellow colour scale indicates the level of the TFCE value’s positive correlation with the MD and RBS scores; areas with significant correlations were identified in the right pre and postcentral regions (A1), prefrontal region in the centre of middle frontal region (widespread from medial to lateral and from ventral to dorsal prefrontal regions including the insula) (A2), parietal lobule (A3), and the left parietal lobule including the inferior parietal lobule (IPL) (A4) and the posterior cingulate region (A5). Residual plots with trend lines depicting correlations between residuals in the multiple regression analyses with RBS scores as the dependent variable and other confounding factors as independent variables; 95% confidence intervals for the trend lines are shown. The peak regional MD values in the key regions in the right precentral region (B1), middle frontal region (B2), IPL (B3), the left parietal lobule (B4), and the posterior cingulate region (B5) are depicted.

We found a significant correlation between the IPS (or RBS) scores and MD using the same analyses described above in the bilateral inferior parietal lobule (IPL), the right prefrontal region in the centre of middle frontal region (from ventral to dorsal and medial to lateral regions including insula) and post- and precentral regions, and the left posterior cingulate region, using the TFCE method with FWE corrected to *P* < 0.0125 (0.05/4) at the whole-brain level (Table 2 and Figure 2).

**Table 2:** Brain regions exhibiting a significant correlation between MD and rule-breaking situation scores.

Brain region	R/L	x	y	z	*TFCE Value*	Corrected *P-*value (FWE)	Cluster size (k_E_)	*β*
Precentral region	R	21	−25.5	63	1185.5	0.011^*^	416	0.099
Postcentral region		10.5	−28.5	75	1169.6	0.011^*^		
Precentral region		18	−19.5	51	1148.7	0.012^*^		
Middle frontal region	R	27	22.5	28.5	1816.4	0.005^*^	11549	0.113
Operculum part of IFR		39	21	16.5	1788.8	0.005^*^		
Middle frontal region		22.5	40.5	13.5	1776.9	0.005^*^		
Precuneus	R	27	−48	28.5	1396.5	0.009^*^	1071	0.125
Parietal operculum		46.5	−33	30	1149.6	0.012^*^		
Superior parietal lobule	L	−22.5	−42	33	1264.7	0.011^*^	1166	0.113
Precuneus		−15	−43.5	39	1206.9	0.011^*^		
Precuneus		−25.5	−61.5	12	1175.1	0.011^*^		
Parietal operculum	L	−45	−24	25.5	1162.1	0.011^*^	349	0.091
Central operculum		−43.5	−15	24	1158.5	0.011^*^		
posterior cingulate region	L	−19.5	−54	7.5	1133.0	0.011^*^	1	0.087

*P < 0.0125, FWE corrected.

FWE: family-wise errors, IFR: inferior frontal region; IPL: inferior parietal lobule, L: left, R: right, MD: Mean diffusivity, TFCE: threshold-free cluster enhancement.

#### Interaction effects of sex and IPS or RBS on brain structures

Using data from both sexes with respect to the covariates of age, RAPM, TIV, and IPS (or RBS) scores, an analysis of covariance (ANCOVA) revealed no significant interaction effect between IPS (or RBS) scores and sex on rWMV, FA, or MD using the TFCE method with FWE corrected to *P* < 0.05 at the whole-brain level.

## Discussion

The present study is the first to investigate direct associations between feelings of guilt and white matter structures in healthy individuals at the whole-brain level. Measures of guilt can be classified into two categories: assessment of states and traits^16^. RBS guilt and IPS guilt seem to be more characteristic traits of guilt rather than the state of guilt using the participant’s answers to questionnaires in many different situations. Importantly and unexpectedly, the right post- and precentral regions were found to be neural correlates of RBS. Consistent with our hypothesized neural correlates for social and moral cognition (IPL including STS and PFC [mPFC, OFC, IFG, and DLPFC]), total RBS scores were positively associated with MD values in the bilateral IPL and the right prefrontal region (widespread from ventral to dorsal and medial to lateral regions including the insula), as well as the left posterior cingulate region.

First, we should outline potential mechanisms by which the post- and precentral regions (sensorimotor regions from arm to hand)^17^ relate to RBS scores. Involvement of the sensorimotor regions in the embodied metaphor of moral-purity is somatotopically organized by linking moral purity with cleansing such as the hands and mouth^18,19^. The ‘Macbeth effect’ addresses a threat to one’s moral purity by cleansing the physical body, especially the hands^20^. Physical cleansing is a psychological mechanism for addressing moral emotions including guilt^20^. Further, the somatosensory cortex is also relevant to the somatic maker hypothesis, which is relevant to the body state structure when the awareness of perceptual signals is necessary for regulating emotions^21^.

Second, there were no significant associations between RBS and other WM measures (rWMV and FA). A plausible reason for this is that DTI results are particularly influenced by WM maturation during adolescence^22^. Young adults in this study might demonstrate higher RBS scores due to developmentally-delayed WM maturation after adolescence. As shown in our previous study^12^, MD and FA measure different microstructural brain properties. MD assesses capillaries, spines, macromolecular proteins, myelin properties, membranes, axons, neuron shapes, protoplasmic and fibrous glia, and enhanced tissue organization^23^. FA is more strongly associated with microstructural properties related to brain connectivity, and is sensitive to increases in axonal membrane thickness, diameter, and/or the extent of parallel organization in axons^23^. Further, FA values are more stable over time (from adolescence to middle age) than MD values^24^. This difference in the degree of change over time might affect sensitivity of FA and MD to detecting regions associated with guilt^12^. For more details, please refer to our previous study^12^.

Third, we should discuss the detected regions in terms of our hypothesis. RBS guilt is more self-oriented than IPS guilt. Notably, the brain’s default mode network (DMN) includes the detected regions, namely the mPFC, PCC, IPL, and precuneus^25^. The DMN, which shows higher levels of activity during passive task conditions than during goal-directed task conditions^25^, has become almost synonymous with self-referential mental activity^26^ in order to meet social cognitive demands^27^. With respect to the PFC, the mPFC is important for reflective self-processing^28^. Guilt has been shown to recruit greater prefrontal regions, a process which may represent demands for behavioural change^29^. Deontological guilt emerges from the internal understanding that one has violated an intuitive moral rule. Punishment is an essential ingredient in deontological guilt because rank differences between people and authority are restored through punishment of the transgressor^30^. The right DLPFC plays a key role in third-party punishment^31^. Based on the somatic marker hypothesis, the vmPFC has an important role in the processes of reasoning and decision making^21^. Defective somatic markers in the vmPFC may serve as a neural framework for explaining immoral or corrupt behaviours^32^. Thus, the vmPFC has an essential role for integrating emotional signals in complex social situations^33^. Further, activity in the vmPFC could link affective disorders to regulation of stressful environments^34^. As for the cingulate cortex, deontological (rule-breaking) moral judgements led to increased activation in the PCC and the right temporoparietal junction^35^. Activity in the PCC was associated with self-relevant moral dilemmas^36^. As we have described previously^37^, regions from the anterior cingulate region to the anterior insula form a salience network, responsible for integrating interceptive information with emotional salience, as in social anxiety. Moreover, deontological guilt selectively activates the insula^38^ and is involved in the processing of disgust and self-reproach^30^. Further, the insula has been shown to encode cognition related to inequity when considering ethical fairness^39^. As described in the introduction, moral reasoning involves a diversity of brain areas including the IPL^11^. The IPL might form a cognitive hub that is uniquely human, and multiple networks converge and interact in the IPL^40^. Interestingly, deontological guilt is involved in obsessive–compulsive disorder (OCD), which is often associated with decreased activation of the ACC and the insula in a clinical fMRI study^41^. Exaggerated guilt-proneness might be relevant to clinical thought-related pathology, such as depression, obsessive compulsive disorders, and paranoia^42^. Greater deontological guilt was related to higher MD (a lower density of neural tissues prohibits free water diffusion), a finding that might relate to diminished moral and social cognition. Decrements to the above-mentioned functions seem to be fundamental of the feeling of deontological guilt.

Fourth, both IPS and RBS scores were higher in females than in males, although no significant gender difference related to the scores was found in regional brain structures. The psychological results were in accordance with the widespread view that women in adolescence through adulthood experience more guilt than men^43^. However, gender differences in guilt feelings tend to be larger among whites than people of other races, in a meta-analysis found^43^. A pilot fMRI study examining neural underpinnings of guilt in a small sample of Germans showed that women activated only temporal regions when experiencing guilt, whereas men showed additional frontal, occipital, and amygdala activation^44^. Further, evidence of gender difference in moral orientation seems to be weak and inconsistent^45^. A study on trait guilt using Japanese undergraduates found no significant gender differences^46^. Thus, the lack of gender differences in neural correlates of guilt feelings might be due to the weak effects of gender differences in morality itself, particularly among individuals who are not white^43^.

Finally, as we have previously noted^47,48^, there are a few limitations to the present study that should be mentioned. Because the present study used a cross-sectional design, the results presented here cannot be used to determine causality between RBS scores and various, associated brain regions. To overcome this limitation, a prospective study that confirms these brain regions are causally necessary to these functions (e.g. a lesion study) remains necessary. Furthermore, we used young, highly-educated, healthy subjects, which may affect generalisability of our findings.

## Methods

### Subjects

The present study included 1196 healthy, right-handed individuals (686 males and 510 females) with a mean age of 20.7 ± 1.8 years. Written informed consent was obtained from each participant prior to beginning the study in accordance with the Declaration of Helsinki (1991). All study procedures were approved by the Ethics Committee of Tohoku University. All experiments were performed in accordance with approved guidelines. For more details regarding procedures undertaken in the present study, please refer to our previous work^49–51^.

### Psychological outcome measures

#### Assessment of feelings of guilt

We used the reliable and valid guilty feeling scale (GFS)^52^, which consists of IPS (11 items) and RBS (10 items) tests^52^. The GFS consisted of 21 statements in total, which were divided into two dimensions. We asked the participants to describe their anticipated feelings towards situations that often elicit feelings of guilt. The four-point rating scale ranged from 1 (no feeling) to 4 (extreme feeling). The internal consistencies (Cronbach’s coefficient of alpha) of the IPS and RBS tests were 0.89 and 0.86^52^, respectively. Convergent validity for the scale was confirmed by significant correlations with other measures (empathy and role-taking ability [social perspective-taking]) related to feelings of guilt. IPS guilt mainly refers to situations in which a friend is hurt, whereas RBS guilt is more public and sometimes involves specific others (parents). For more details, please refer to our previous study^7^.

#### Psychometric measures of general intelligence

The RAPM, which is a widely used measure of general intelligence^53^, was utilized in the present study. This measure was adjusted to assess the effects of general intelligence on brain structures^54–56^.

#### Behavioural data analyses

All behavioural data were analysed using the IBM SPSS Statistics 22.0 software package (IBM Corp.; Armonk, NY, USA). Differences between males and females in terms of age and scores on the cognitive measures (RAPM, the IPS, and the RBS scores) were analysed by one-way ANOVA; a two-tailed *P* value < 0.05 was considered to indicate statistical significance.

### Image acquisition

#### Structural MRI

All MRI data were acquired using a 3T Philips Achieva scanner (Philips Medical Systems, Best, Netherlands). Three-dimensional high-resolution T1-weighted images were collected using a magnetisation-prepared rapid gradient-echo sequence with the following parameters: 240 × 240 matrix, TR = 6.5 ms, TE = 3 ms, TI = 711 ms, FOV = 24 cm, 162 slices, in plane resolution = 1.0 × 1.0 mm, slice thickness = 1.0 mm, and a scan duration of 483 s.

Diffusion-weighted data were acquired using a spin-echo echo-planar imaging (EPI) sequence with the following parameters: TR = 10293 ms, TE = 55 ms, FOV = 22.4 cm, 2 × 2 × 2 mm^3^ voxels, 60 slices, SENSE reduction factor = 2, and number of acquisitions = 1. Diffusion weighting was isotropically distributed in 32 directions (*b* value = 1,000 s/mm^2^) and three images with no diffusion weighting (*b* value = 0 s/mm^2^; b = 0 images) were acquired using the spin-echo EPI sequence (TR = 10293 ms, TE = 55 ms, FOV = 22.4 cm, 2 × 2 × 2-mm^3^ voxels, 60 slices). Acquisitions for phase correction and signal stabilization were performed, though these data were not used for image reconstruction. For more details regarding these procedures, please refer to our previous work^23,47,57^.

### Pre-processing and analyses of structural data

#### VBM data

All pre-processing of the T1WIs data was performed using Statistical Parametric Mapping software (SPM8; Wellcome Department of Cognitive Neurology, London, UK) according to the protocol described for VBM analyses in a previous report from our group^58^. Using the new segmentation algorithm implemented in SPM8, T1-weighted structural images from each individual were segmented and normalized to Montreal Neurological Institute (MNI) space to yield images with 1.5 × 1.5 × 1.5-mm voxels using diffeomorphic anatomical registration through the exponentiated lie algebra (DARTEL) registration process implemented in SPM8. In addition, we performed a post-hoc volume change correction (modulation)^59^. All images were smoothed by convolving them using an isotropic Gaussian kernel of 6 mm full-width at half maximum (FWHM). For additional details, please refer to our previous work^58,60^.

#### FA and MD data

All pre-processing and analyses of the imaging data were performed using SPM8 implemented in MATLAB (MathWorks Inc.; Natick, MA, USA). The methods and parameters were optimized and validated using SPM8, whereas those using SPM12 were not validated in our previous study^47^. Normalized FA images were then masked by a custom mask image most likely to be white matter, then smoothed by a Gaussian kernel of 8 mm FWHM. These methods were adapted from a previous study^37^.

The MD map was calculated from the collected images using a commercially available diffusion tensor analysis package (Philips Medical Systems, Best, Netherlands), run on the MR console. These procedures involved corrections for motion and distortion caused by eddy currents, and all calculations were performed using a previously described method^61^. Briefly, the MD images from participants were normalized using a previously validated DARTEL-based registration process to develop images with 1.5 × 1.5 × 1.5 mm voxels^61^. Next, tissues that were least likely to be grey or white matter were manually removed and the images were smoothed by convolving them using an isotropic Gaussian kernel of 8-mm FWHM. For additional details, please refer to our previous work^47,48^.

As in our previous study^47^, we did not and could not utilize tract-based spatial statistics (TBSS)^62^. We instead used SPM-based analyses and believe that our new DTI preprocessing methods substantially alleviate the problems associated with voxel-based DTI analyses^62^. Further, TBSS uses only the skeleton of an FA map, with the result that voxels containing peripheral white matter are not included. Hence, methodological improvements to TBSS are needed to increase its reliability^63^. For more details, please refer to our previous study^47^.

### Statistical group-level analyses of imaging and behavioural data

Correction for multiple comparisons was performed using TFCE^64^ with randomised (5,000 permutations) nonparametric testing in the TFCE toolbox (http://dbm.neuro.uni-jena.de/tfce/). A FWE corrected threshold of *P* < 0.0125 (0.05/4) was applied. For more details, please refer to a previous study by our group^37^.

#### VBM data

A whole-brain multiple regression analysis was performed in SPM8 and used to assess the association between rWMV and IPS (or RBS) scores. Covariates for this analysis included sex, age, RAPM score, and TIV. For each covariate, the overall mean was used for mean centring. Analyses for rWMV were performed in voxels for all subjects that showed a signal intensity >0.05.

We also investigated whether the relationship between rWMV and guilt scores differed between males and females at the level of the whole brain. We employed a voxel-wise ANCOVA in which sex difference was a group factor, using *t*-contrasts (using the full factorial option of SPM8). In this analysis, age, RAPM, TIV, and IPS (or RBS) scores were modelled to enable detection of unique relationships with rWMV (using the interaction option in SPM8 for each sex). TIV was modelled to have a common relationship with rWMV across both sexes.

#### FA and MDdata

A voxel-by-voxel regression analysis was performed in SPM8 using the FA or MD value at each voxel as the dependent variable and age, sex, RAPM score, and IPS (or RBS) scores as independent variables. The analysis using FA was limited to areas within the white matter, whereas the analysis using MD was limited to areas within the grey and white matter masks that were created using the procedures described above. We also investigated whether the relationship between FA or MD and IPS (or RBS) scores differed between men and women by using the same method as in rWMV.
